# Microglia in autism spectrum disorder: heterogeneity, immunometabolism, and synapse-related pathways

**DOI:** 10.3389/fimmu.2026.1783755

**Published:** 2026-04-28

**Authors:** Aojie Lian, Mei He, Hong Zhang, Yingmei Yang

**Affiliations:** 1 NHC Key Laboratory of Birth Defect for Research and Prevention, Hunan Provincial Maternal and Child Health Care Hospital, Changsha, Hunan, China; 2 Hunan Provincial University Key Laboratory of the Fundamental and Clinical Research on Neurodegenerative Diseases, Changsha Medical University, Changsha, China; 3 The Third Xiangya Hospital of Central South University, Changsha, China

**Keywords:** astrocyte-microglia crosstalk, autism spectrum disorder, complement, developmental timing, immunometabolism, microglia, synaptic pruning, TSPO PET

## Abstract

Microglia are increasingly implicated in autism spectrum disorder (ASD), but their role remains difficult to define because the available evidence is heterogeneous in cohort composition, developmental stage, sampled brain region, and experimental modality. This Review summarizes current evidence on three related aspects of ASD-relevant microglial biology: microglial heterogeneity, immunometabolic regulation, and synapse-related pathways. Human postmortem studies, bulk transcriptomics, single-cell and spatial atlases, methylomic deconvolution, and *in vivo* neuroimmune imaging collectively support the presence of immune- and glia-associated alterations in at least a subset of ASD brains, but these findings do not support a single ASD-wide microglial phenotype. Instead, current evidence is more consistent with region-, stage-, sex-, and context-dependent microglial variation that should be interpreted together with neuronal, astrocytic, vascular, and broader tissue-level changes. We further review how lipid handling, mitochondrial function, phagocytic-lysosomal load, and bioactive lipid signaling may influence microglial competence in ASD-relevant settings, while noting that much of the detailed mechanistic immunometabolism literature still derives from aging and neurodegeneration. At the microglia-synapse interface, complement deposition, phosphatidylserine exposure, anti-engulfment checkpoints, and astrocyte-microglia crosstalk provide more informative mechanistic entry points than broad activation terminology. Across studies, the major challenge is not whether microglia are involved in ASD, but how to distinguish primary pathogenic effects from secondary adaptation, and how to relate molecular signatures to excessive, insufficient, or mistargeted synaptic remodeling. Overall, the literature supports a more precise interpretation of ASD-related microglial biology based on developmental timing, cellular context, and mechanism-linked readouts rather than non-specific inflammatory labels alone.

## 1 Introduction

Autism spectrum disorder (ASD) is a heterogeneous neurodevelopmental condition characterized by early-appearing differences in social communication together with restricted and repetitive behaviors, with substantial variability in clinical presentation, developmental course, and genetic architecture (1). Work across human genetics, postmortem transcriptomics, and experimental systems has increasingly suggested that ASD risk converges on biological processes related to synapse development, circuit maturation, and cell-type-specific regulation of brain development rather than on a single pathogenic pathway (2). At the molecular level, several postmortem cortical studies have reported a recurring synaptic-immune pattern, with reduced neuronal or synaptic gene expression alongside increased immune- and glia-associated signals in at least a subset of ASD brains (3) (4). Taken together, these observations have sustained interest in whether neuroimmune processes, including microglial responses, contribute to atypical circuit development in ASD.

Microglia are the resident myeloid cells of the central nervous system and play central roles in brain development and homeostasis. They respond to neuronal activity, shape neurogenesis, monitor tissue integrity, and participate in synaptic remodeling through mechanisms that include complement-linked tagging, phagocytic clearance, and contact-dependent remodeling (5–7). At the same time, microglia do not operate in isolation. Astrocytes also participate directly in synapse elimination, and microglia-astrocyte interactions can reshape inflammatory tone, synaptic support, and pruning-related pathways (8, 9). These considerations make it increasingly difficult to interpret “microglial” signals in ASD without embedding them in a broader multicellular context that includes neurons, astrocytes, oligodendroglial populations, and the neurovascular niche.

Recent cell-resolved and spatial studies have further reinforced that ASD-associated molecular alterations are distributed across multiple neural and glial compartments, including microglia, and that these changes are strongly context dependent rather than reducible to a single broad inflammatory or reactive state (10, 11). This shift has important implications for ASD research, where diverse findings are still often compressed into non-specific labels such as “activation” or “neuroinflammation,” despite the fact that such terms can blur distinctions among altered cell composition, context-dependent phenotypic heterogeneity, secondary responses to neuronal dysfunction, and pathway changes that may have more specific relevance to synaptic remodeling.

A central challenge is that most human ASD evidence remains cross-sectional and largely postmortem (12–15). By contrast, *in vivo* neuroimmune imaging readouts such as TSPO PET provide glia-associated rather than microglia-specific signals (16–18). Interpretation is further shaped by effect modifiers that are often unevenly represented or incompletely reported, including developmental stage, sex, brain region, epilepsy and other comorbidities, medication exposure, psychiatric subgroup structure, and perimortem factors. For this reason, no single modality can currently define a universal ASD microglial phenotype.

Several recent developments make a careful reassessment timely. Human single-nucleus RNA-seq, chromatin accessibility, methylomic deconvolution, and spatial transcriptomic datasets now provide cell-type-resolved evidence for ASD-associated molecular changes across multiple neural and glial compartments, including microglia (10, 11, 19–21). In parallel, ASD-relevant experimental systems and functional genomic studies have strengthened interest in pathways linking microglial variation to synaptic remodeling, immune signaling, and metabolic adaptation (22–24). At the same time, growing attention to immunometabolism has highlighted lipid handling, mitochondrial fitness, phagocytic-lysosomal load, and bioactive lipid signaling as candidate regulators of microglial function, while also underscoring that much of the detailed mechanistic literature in this area comes from aging and neurodegeneration rather than ASD itself.

In this Review, we synthesize current evidence on microglial heterogeneity, immunometabolism, and microglia-synapse interfaces in ASD, with emphasis on where findings converge, where they remain context dependent, and where mechanistic interpretation remains premature. We focus on three related questions. First, what forms of microglial heterogeneity recur across ASD-relevant studies, and how should they be described without collapsing them into one generic activation spectrum? Second, which immunometabolic processes have meaningful support in neurodevelopmental and ASD-relevant contexts, and which remain extrapolated largely from other disorders? Third, which synapse-related pathways provide the most plausible links between glial alterations and circuit-level outcomes in ASD? Throughout, the aim is to synthesize prior evidence, clarify inferential limits, and strengthen narrative integration across modalities rather than to advance a single unifying causal model.

## 2 Scope of the review and literature identification

**Figure 1:**
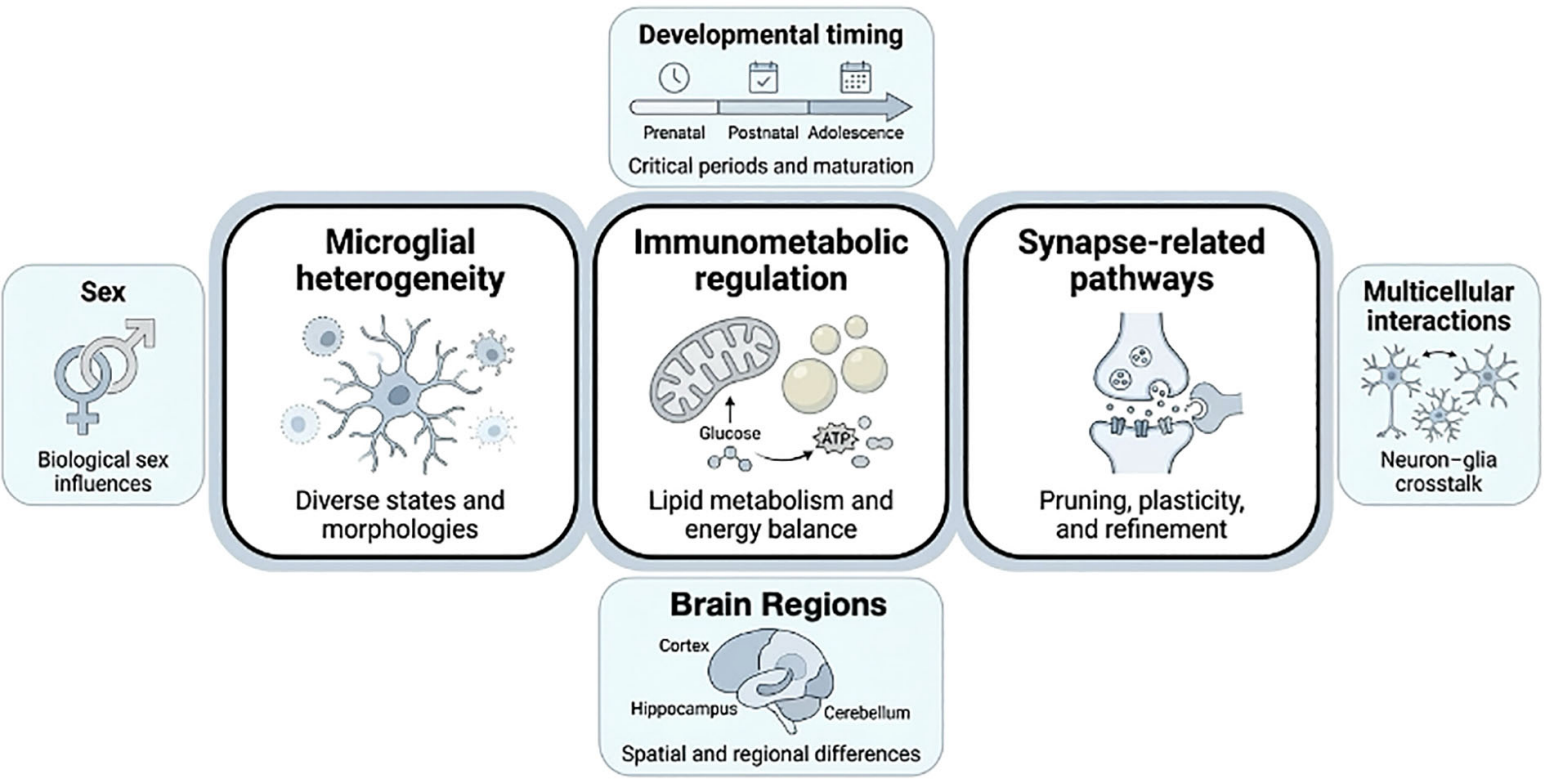
Overview of the review focus. The figure highlights three central themes recurrent in the ASD microglia literature: microglial heterogeneity, immunometabolic regulation, and synapse-related pathways. These themes are interpreted in the context of developmental timing, brain region, biological sex, and multicellular interactions, all of which may shape the emergence, interpretation, and functional relevance of microglial alterations in ASD.

**Figure 2:**
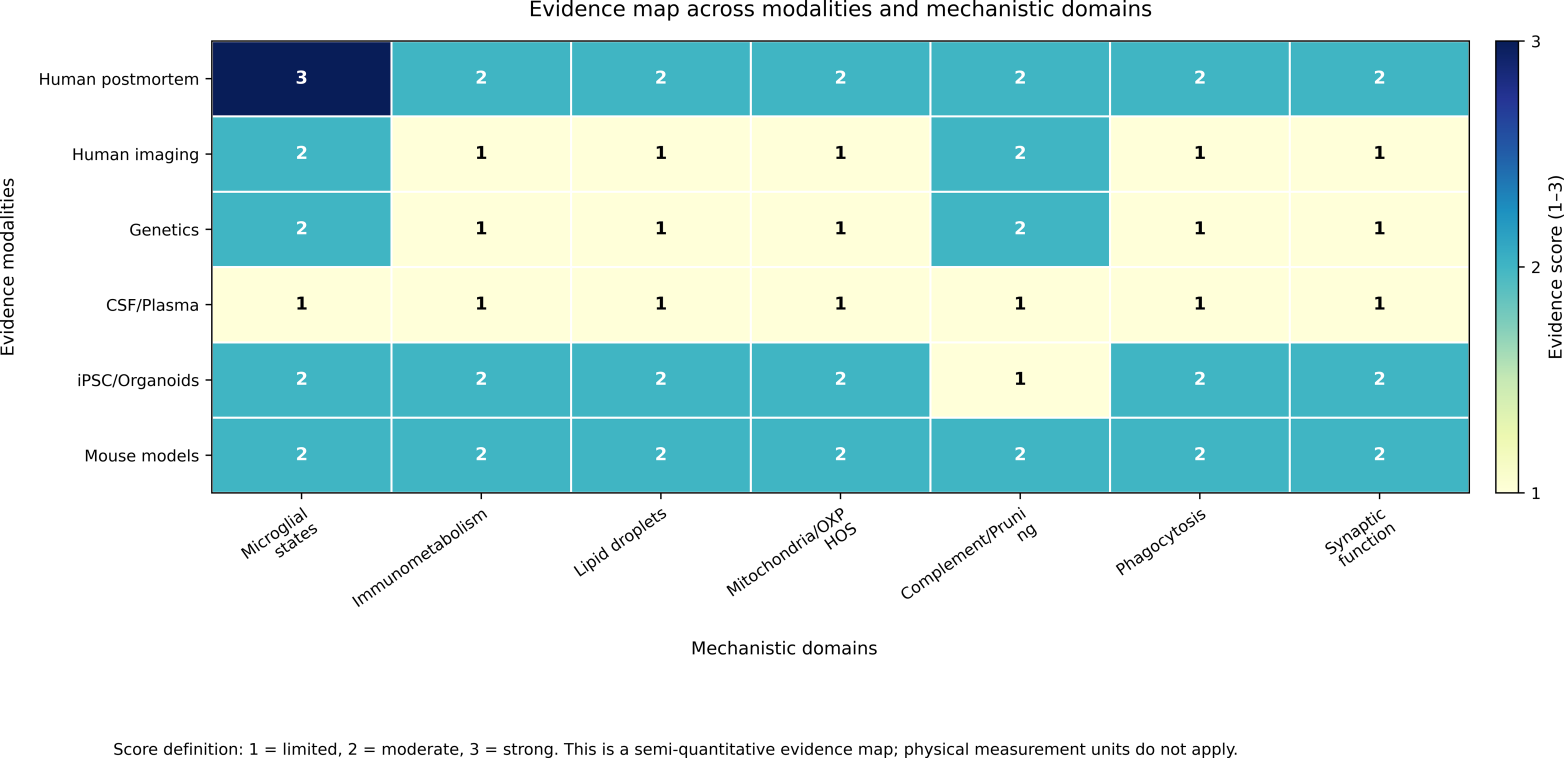
Evidence map across modalities and mechanistic domains. Semi-quantitative evidence strength is summarized across major modalities and mechanistic domains discussed in this Review, including postmortem histology, bulk transcriptomics, single-cell and spatial profiling, methylomic deconvolution, *in vivo* neuroimmune imaging, and ASD-relevant experimental systems. Scores reflect confidence in the literature base rather than effect size and integrate human relevance, replication or convergence, mechanistic specificity, and experimental support.

Literature discussed in this Review was identified through targeted searches of PubMed and Google Scholar through January 2026 using combinations of terms related to autism spectrum disorder, microglia, neuroinflammation, immunometabolism, synaptic remodeling, complement, astrocytes, and neuroimmune imaging. These searches were supplemented by citation chaining from primary ASD studies, relevant methodological papers, and recent reviews. Because this article is a narrative Review rather than a formal systematic review or meta-analysis, the text emphasizes recurrent findings, major areas of convergence, and key interpretive limitations. **Figure 1** provides an overview of the review focus and its three central themes: microglial heterogeneity, immunometabolic regulation, and synapse-related pathways. **Figure 2** summarizes the current evidence base across major modalities and mechanistic domains discussed in this Review. Additional search strings, study prioritization details, and the evidence organization workflow are provided in Text S1 and Supplementary **Figure 1** of the Supplementary Material.

## 3 Human evidence linking microglia to ASD

Human evidence linking microglia to ASD comes from postmortem histology and biochemistry, bulk and cell-resolved transcriptomics, methylomic deconvolution, and *in vivo* neuroimmune imaging. A summary metadata table of primary ASD microglia-relevant studies is provided in **Table 1**, and an expanded study-level evidence summary is provided in **Supplementary Table 1** of the Supplementary Material.Considered together, these studies support the view that immune- and glia-associated abnormalities occur in at least a subset of ASD brains. At the same time, the same literature also shows why it remains difficult to infer a single ASD-wide microglial phenotype from any one modality. A modality-specific summary of common confounders, practical safeguards, and interpretation notes is provided in **Supplementary Table 2** of the Supplementary Material.

**Table 1:** Summary metadata of ASD microglia-relevant evidence discussed in this Review.

Evidence type	Study (Author, Year)	System / cohort / model	Microglia-related readout(s)	Key ASD-relevant microglia point	Brief note
Human postmortem transcriptomics	Voineagu, 2011	Postmortem human brain	Co-expression modules (asdM16); A2BP1/FOX1 splicing	Identified asdM16 module enriched in glial markers linked to transcriptional dysregulation	PMI, RIN, age, and medication history noted
Human postmortem transcriptomics	Gupta, 2014	Postmortem human brain	Co-expression (mod5); type I IFN response genes	Pinpointed asdM16 to innate immunity and M2-state microglial response	PMI, RIN, and brain bank source noted
Human postmortem transcriptomics	Velmeshev, 2019	Human postmortem snRNA-seq	Activation markers; developmental TFs (AHI1)	ASD microglia were enriched for activation genes and dysregulated developmental TFs	Clinical severity correlations reported
Human postmortem transcriptomics	Wamsley, 2024	Human postmortem snRNA/TAC	Glia-reactivity GRNs (IRF8); MG2 state proportion	Identified reactive MG2 cluster significantly increased in ASD frontal cortex	Included dup15q (15q11–13) monogenic cases
Human in vivo imaging	Suzuki, 2013	Human 11C-PK11195 PET	11C-PK11195 binding potential (BP)	First in vivo evidence of augmented microglial activation	IQ-matched high- and low-IQ subsets
Human in vivo imaging	Zürcher, 2021	Human 11C-PBR28 MR-PET	TSPO regional distribution (SUVR)	Reported lower regional TSPO expression, suggesting a distinct neuroimmune profile in adult males	TSPO genotype correction applied
Human in vivo imaging	Tseng, 2024	Human 11C-PBR28 PET-MRI pilot	11C-PBR28 binding potential (BP)	Female pilot study showing regional TSPO elevation in MOC and splenium	Menstrual cycle phase monitoring noted
Animal model	Guneykaya, 2023	Neuroligin-4 KO mouse (C57BL/6J)	Proteomics, phagocytosis, P2RY12 current	Identified a male-specific microglial state with metabolic and phagocytic impairments	17β-estradiol rescue effect in males
Animal model	Dalton, 2024	C58/J mouse (sociability focus)	snRNA-seq; homeostatic markers (Trem2, Cx3cr1)	Linked sociability deficits to reduced microglial homeostatic marker expression	Strain differences (C58/J vs C57BL/6J)
In vitro functional genomics	Teter, 2025	CRISPRi screen in human hiMG	FACS-based synaptic pruning; endocytosis	Identified ADNP as a key modifier of microglial synaptic pruning and endocytosis	In vitro iTF-Microglia system limitations
Review / perspective	Trujillo Villarreal, 2021	Narrative synthesis / conceptual review	Not applicable	Primed microglia in obesity may disrupt mesocorticolimbic circuits	Focus on dopamine circuit E/I balance
Mouse model + organoid	Wu, 2024	SCN2A-deficient model	Spine density; microglial over-pruning; C3 cascade; PLX3397 ablation	Microglial over-pruning of post-synapses via the C3 cascade led to reduced transmission	PLX3397 microglial ablation reversed phenotype
Animal model	Tian, 2024	VPA rat model (prenatal exposure)	TREM2/DAP12; p38 MAPK pathway	TREM2 improved synaptic development by inhibiting p38 MAPK signaling	Supported by in vitro rat primary microglia validation
Animal model	Wang, 2025	Microglial TREM2 deficiency model	Kv1.3 activity; mEPSCs/mIPSCs; TNF-α/IL-1β	TREM2 deficiency increased Kv1.3 activity, causing E/I imbalance and hyperactivity	Pharmacological Kv1.3 inhibition rescue (PAP-1)
Animal model	Lu, 2025	TREM2 knockdown (AAV)	Iba1-positive/CD68 markers; RA/RARα signaling	Disruption of TREM2–RA/RARα signaling led to aberrant microglial synaptic pruning	Oral RA supplementation rescue effect
Review / perspective	Boller, 2025	Narrative synthesis / perspective	Not applicable	CACNA1C genetic risk may interact with adaptive immune memory in neuroinflammation	Focus on L-type Ca2+ channels (CaV1.2)
Review / perspective	Bilbo, 2018	Narrative synthesis / perspective	Not applicable	Non-infectious MIA may trigger microglial priming and ASD risk	Focus on the “multiple-hit” hypothesis
Review / perspective	Maldonado-Ruiz, 2019	Narrative synthesis / perspective	Not applicable	Maternal nutritional programming may program central microglial activation in MIA contexts	Links obesity-related exposures to synaptic inputs
Animal model	Wang YC, 2023	HFD × Cx3cr1 cKO mouse	Iba1-positive cell density; activated microglial number	High-fat diet exacerbated ASD-like behavior via CA1 microglial activation	Minocycline treatment reversed behavioral deficits
Human postmortem transcriptomics	Mou, 2022	Human SVZ postmortem study	C4 mRNA/protein; Ki67 cell density	Linked complement C4 to neuroimmune abnormalities and neurogenesis changes in the SVZ	SVZ-specific focus; PMI and RIN controlled
Review / perspective	Jántti, 2026	Narrative synthesis / perspective	Not applicable	Astrocyte-microglia crosstalk may regulate E/I balance via mTOR and complement	Focus on human-relevant iMGL models
Review / perspective	Lyamtsev, 2025	Narrative synthesis / perspective	Not applicable	Male microglia may be more prone to pro-inflammatory responses, favoring early ASD manifestation	SVZ-specific focus; PMI and RIN controlled
Animal model	Zhang Q, 2024	VPA-induced mouse model (C57BL/6)	snRNA-seq; TNF/NF-κB pathways	Males showed more significant upregulation of inflammatory pathways in hippocampal microglia	45,693 single nuclei analyzed across 12 cell types

This table provides a condensed summary of the evidence types, systems, readouts, principal ASD-relevant microglia findings, and brief notes for the studies and evidence sources included in **Supplementary Table S1**.Note: This table is intended as a condensed summary corresponding to **Supplementary Table S1**.

Early postmortem studies reported increased cytokines, chemokines, and glial reactivity markers in ASD cortex and cerebellum, bringing neuroimmune mechanisms into the center of ASD discussion (12). Subsequent histological work described altered microglial density, morphology, and microglia-neuron spatial relationships in selected cortical regions (13), as well as region- and layer-dependent variation in microglial markers across ASD postmortem tissue (14). Additional transcript-level studies likewise reported inflammation-related and glia-associated signatures in ASD cortex (15). These studies were important in establishing that microglia deserve attention in ASD, but they also had predictable limitations, including modest cohort sizes, heterogeneous ages, variable control for epilepsy and other comorbidities, incomplete medication histories, and limited ability to distinguish altered microglial function from broader tissue pathology.

Bulk cortical transcriptomic studies later reinforced the general picture by identifying recurrent immune- and glia-associated upregulation together with downregulation of synaptic and neuronal gene modules in ASD cortex (3, 4). These findings remain influential because they suggest convergent coupling between synaptic dysregulation and neuroimmune change. At the same time, bulk tissue averages cannot separate within-cell molecular change from shifts in cell composition, and they are sensitive to region, developmental stage, RNA quality, agonal factors, and donor structure. Broader cross-disorder analyses further indicate that some immune and glial signals are shared across psychiatric disorders, which strengthens their biological interest but also cautions against interpreting them as ASD-specific in a simple categorical sense (25, 26).

Cell-resolved approaches have improved the resolution of this literature. Single-nucleus RNA-seq studies in human cortex have shown that ASD-associated transcriptional changes are distributed across multiple neuronal and glial cell types, including microglia, rather than being restricted to a single immune compartment (10). More recent large-scale single-nucleus and spatial profiling studies have supported the presence of region- and donor-dependent reactive glial alterations in ASD cortex, within a multicellular context that includes excitatory neurons, interneurons, astrocytes, oligodendroglial populations, and vascular-associated cells (11). Additional human studies have expanded the anatomical scope of ASD microglia-related evidence. Single-nucleus analysis of the early postnatal amygdala revealed cell-type-specific ASD-associated transcriptional changes in a region central to social and affective processing (19). Complement-related neuroimmune abnormalities were also reported in the subventricular zone and adjacent tissue in ASD brains (20). At another level, methylomic deconvolution across large postmortem cohorts suggested increased microglial representation in autism, indicating that some ASD findings may reflect altered cellular composition in addition to cell-intrinsic molecular change (21).

Taken together, these transcriptomic and cell-resolved studies support several conclusions. First, immune- and glia-associated changes in ASD are not simply artifacts of one cohort or one modality. Second, the relevant biology is distributed across multiple cellular compartments rather than confined to microglia alone. Third, the value of cell-resolved and spatial approaches lies not in establishing one canonical ASD microglial state, but in making it possible to distinguish composition effects from cell-intrinsic shifts and to localize glial alterations to defined anatomical and cellular contexts.

*In vivo* neuroimmune imaging provides a different but complementary layer of evidence. TSPO PET has been used to examine glia-associated neuroimmune signals in living individuals with ASD, but interpretation is complex because TSPO is not microglia specific and because ligand binding, mitochondrial biology, genotype, psychiatric subgroup structure, and kinetic modeling all influence the signal (16) (17, 18). Published ASD studies have not yielded a single consistent direction. A first-generation tracer study reported increased regional TSPO signal in young adults with ASD (27). In contrast, a later [^11C]PBR28 MR-PET study reported lower regional binding in young adult males with ASD (28). A subsequent [^18F]FEPPA study with kinetic modeling found no significant case-control difference in the primary analysis, but suggested lower binding after excluding ASD participants with current major depressive episodes, illustrating the importance of psychiatric subgroup structure (29). A more recent pilot study in females suggested that sex may further modify TSPO findings in ASD (30).

These apparently discordant findings are not necessarily contradictory. Postmortem molecular and histological studies capture terminal tissue biology shaped by region, developmental history, disease duration, cause of death, agonal factors, RNA quality, and treatment exposure. By contrast, TSPO PET measures a living glia-associated signal that is influenced by genotype, tracer characteristics, kinetic modeling, mitochondrial biology, and psychiatric comorbidity. The two modalities therefore sample different biological layers. A lack of one-to-one agreement between postmortem immune signatures and TSPO PET should not be interpreted as evidence against microglial involvement in ASD. Rather, it indicates that current human evidence is heterogeneous and only partially identifiable.

Overall, the human literature supports several restrained conclusions. Immune- and glia-associated abnormalities are reproducibly observed in at least a subset of ASD brains. These abnormalities are better interpreted as heterogeneous and context dependent than as evidence for a single uniform microglial phenotype. Cell-resolved studies have made the field more informative by showing that microglial findings in ASD arise within broader multicellular pathology rather than in isolation. Finally, discrepancies between postmortem and *in vivo* studies reflect the fact that these modalities interrogate different biological layers and are influenced by different sources of variation. These points set the stage for a more careful discussion of microglial heterogeneity in ASD.

## 4 Microglial heterogeneity in ASD

### 4.1 Limits of broad activation labels in ASD microglia research

Microglial heterogeneity has become a central topic across neurobiology, and this broader shift is directly relevant to ASD. In earlier literature, microglial findings were often summarized using broad terms such as activation, reactivity, or neuroinflammation. These terms remain useful as general descriptors, but they are often insufficient for interpreting cell-type-resolved ASD data because they do not distinguish altered cell abundance, lysosomal burden, interferon signaling, lipid handling, phagocytic competence, oxidative stress responses, or other context-specific features. Recent single-cell atlases have instead shown that microglia vary across developmental stage, anatomical niche, and disease context, with no universal binary division that can adequately capture this diversity (31) (32, 33). In this Review, terms such as state and program are used as descriptive analytic terms rather than as formal taxonomic labels. A literature-oriented summary of recurrent microglia-related states, pathways, and functional themes discussed across ASD studies is provided in **Supplementary Table 3** of the Supplementary Material.

This broader field-wide shift is useful for ASD, but ASD-specific evidence should remain the starting point for interpretation. In ASD studies, early postmortem histology and cortical transcriptomics already suggested that immune- and glia-associated abnormalities recur in at least a subset of brains, although these reports could not resolve whether the dominant signal reflected microglial state change, altered cell composition, substrate burden, or broader tissue pathology (3, 4, 12–15). The main implication is therefore not that ASD already has a settled microglial taxonomy, but that broad activation language is too coarse to capture the biological variation now visible across donors, regions, and modalities.

It is also important to distinguish recurrent pathway modules from stable cell identities. Interferon-related, phagocytic-lysosomal, complement-associated, oxidative stress, and lipid-handling signatures may be engaged to different degrees across multiple microglial phenotypes rather than defining one fixed ASD microglial “type” (34). That distinction is particularly important in ASD, where most human data remain cross-sectional and where composition effects, developmental timing, sex, and regional sampling can all influence how a microglial signal appears.

### 4.2 Recurrent patterns across ASD-relevant studies

When ASD studies are considered together, several recurrent biological themes emerge. Bulk cortical datasets repeatedly identify a synaptic-immune pattern, with reduced neuronal or synaptic gene expression together with increased immune- and glia-associated signals in subsets of ASD cortex (3) (4). Cell-resolved human datasets extend this observation by showing that ASD-associated changes involve multiple neuronal and glial compartments, including microglia, rather than one isolated immune compartment (10, 11, 19). Regionally focused work further broadens the ASD context: complement-linked neuroimmune abnormalities have been reported in the subventricular zone (20), and methylomic deconvolution across large postmortem cohorts supports increased microglial representation in autism at the level of cell-type composition (21).

Across these ASD-relevant studies, the recurring themes are narrower than the full vocabulary of the broader microglia field. One theme involves inflammatory, interferon-related, or stress-associated signaling. A second involves lysosomal, phagocytic, and complement-related machinery. A third involves lipid-handling and metabolism-related pathways. These themes do not yet define one shared ASD microglial state, but they do justify a literature-based discussion of recurrent mechanistic dimensions across ASD studies.

Experimental systems linked to ASD genetics are beginning to reinforce the same broad axes. In the Neuroligin-4 knockout model, microglia displayed sex-specific changes in metabolism and functional profile, indicating that microglial alterations in ASD-related models may be conditional rather than uniform (22). Transcriptomic studies in the C58/J amygdala model also reported sex-dependent glial differences involving microglia and oligodendroglial populations (23). In SCN2A-deficient mice and human cerebral organoids, microglia-dependent over-pruning was linked to ASD-relevant neuronal perturbation (35). In parallel, CRISPRi-based interrogation of ASD risk genes in human microglia showed effects on endocytosis and synaptic pruning, providing a route from autism genetics to testable microglial mechanisms (24). Together, these studies do not establish a single autism-specific microglial identity, but they do support the view that ASD-related microglial variation is better described through pathway-level and function-linked differences than through broad activation labels.

### 4.3 Human cell-resolved evidence and multicellular context

Human cell-resolved evidence now extends beyond the general observation that ASD brains show glia-associated abnormalities. In cortex, single-nucleus studies identified cell-type-specific molecular changes involving microglia alongside excitatory neurons, interneurons, astrocytes, and oligodendroglial populations (10, 11). In the early postnatal amygdala, single-nucleus analysis likewise revealed cell-type-specific ASD-associated transcriptional changes in a region central to social and affective processing (19). Complement-associated abnormalities in ASD have also been described in the subventricular zone and adjacent regions (20). At another level, methylomic deconvolution across large postmortem cohorts suggests that autism can involve cell-type compositional shifts, including increased microglial representation, rather than only within-cell transcriptional change (21).

These studies strengthen several points. First, ASD-associated molecular alterations are distributed across multiple cell populations, and microglial findings are best interpreted within that broader multicellular context. Second, the human evidence is compatible with both within-cell microglial shifts and altered microglial abundance or composition. Third, even cell-resolved human data do not by themselves determine whether the observed microglial signal is primary, secondary, adaptive, or mixed, nor do they specify the functional direction of synapse-related change.

For this reason, the strongest current conclusion is not that a single ASD microglial phenotype has been established, but that human ASD studies now support reproducible, context-dependent microglial involvement that varies with region, donor structure, developmental stage, and modality. This conclusion is more consistent with the current evidence than either broad activation language or premature taxonomic claims.

### 4.4 Comparison with broader microglia literature and the place of SPP1-linked signatures

ASD literature has sometimes borrowed microglial labels from other disorders without sufficient attention to biological context. This is especially relevant for lipid-phagocytic or SPP1-linked signatures, which are now frequently discussed across neurodegeneration and cross-disease human atlases. Mechanistic work in neurodegeneration shows that SPP1 can be associated with phagocytic-state induction and synaptic engulfment under defined pathological conditions (36). More recently, cross-condition human microglia atlasing has incorporated ASD together with other neurological disorders, providing a useful reference framework for comparing recurrent subsets across diseases without assuming that similarly named populations are biologically identical (37).

In ASD itself, however, the evidence is more limited and more indirect. Wamsley et al. identified reactive glial alterations involving microglia and astrocytes in human ASD cortex (11), and Teter et al. used postmortem ASD microglial differential-expression signatures as a human reference when interpreting functional genomic perturbations in human microglia (24). These studies make lipid-handling and phagocytic programs relevant to ASD discussion, but they do not justify declaring a universal SPP1-positive or disease-associated microglial identity across ASD donors or regions.

The most appropriate use of SPP1-linked and related signatures in ASD literature is therefore comparative. They can help frame questions about whether some ASD-associated microglial shifts overlap with lipid-handling, lysosomal, or phagocytic phenotypes and whether such overlap corresponds to measurable complement deposition, engulfment burden, or lysosomal throughput. Used in this way, these signatures remain evidence-based comparison points rather than stand-alone taxonomic conclusions.

Overall, current human and experimental data justify several conclusions, but not more than that. They justify saying that microglia are involved in ASD-related brain pathology, that their molecular profiles vary across donors and regions, and that these profiles overlap with phagocytic, lysosomal, complement-associated, inflammatory, and metabolic pathways. They do not yet justify defining one ASD-wide microglial phenotype or assuming that a label imported from another disorder carries identical meaning across cortex, amygdala, subventricular zone, organoid systems, and experimental models. Maintaining this distinction helps keep interpretation anchored in ASD-specific evidence while still allowing comparison with the broader microglia literature.

## 5 Immunometabolism in ASD-relevant microglia

Immunometabolism refers to the reciprocal relationship between immune function and cellular metabolism, whereby metabolic pathways shape immune behavior and immune activation reshapes metabolic demand (38). In microglia, this concept has become increasingly relevant because cellular functions most pertinent to brain development and disease, including process motility, chemotaxis, engulfment, lysosomal degradation, and cytokine production, all depend on energetic state and substrate availability. For ASD, immunometabolism offers an important interpretive layer because it helps explain how microglia with superficially similar inflammatory signatures may differ in actual functional competence.

A key issue, however, is that much of the detailed mechanistic literature on microglial immunometabolism comes from aging and neurodegeneration rather than from neurodevelopment or ASD. This creates both an opportunity and a limitation. The opportunity is that lipid metabolism, mitochondrial regulation, lysosomal load, and bioactive lipid signaling are now well-established determinants of microglial behavior in other contexts. The limitation is that these findings cannot be transferred to ASD without considering developmental stage, tissue substrate, and disease context. A careful review therefore needs to use this literature for mechanistic guidance while keeping ASD-specific evidence at the center of interpretation.

### 5.1 Lipid handling and receptor-linked metabolic support

Lipid handling is a prominent example of the link between metabolism and immune function in microglia. Microglia continuously process lipid-rich substrates derived from myelin, apoptotic material, and synaptic components, and genes involved in cholesterol transport, lipoprotein metabolism, fatty acid handling, and lipid storage recur across many microglial phenotypes. ABCA1-dependent ApoE lipidation is one relevant pathway, because disruption of ABCA1 alters ApoE secretion and lipid homeostasis in glial cells, including microglia (39). Reviews of microglial lipid and lipoprotein metabolism have further emphasized the recurrent involvement of ApoE-, TREM2-, and lysosome-related pathways in shaping microglial responses across development, injury, and disease (40, 41).

Among these pathways, TREM2 is especially informative because it links receptor signaling to metabolic competence. In neurodegeneration, TREM2 deficiency impairs microglial energetic and biosynthetic metabolism and weakens mTOR-dependent support for microglial function (42). This evidence was established outside ASD, but it provides a concrete framework for considering how impaired lipid sensing or metabolic support could constrain microglial function more generally.

More importantly, direct neurodevelopmental anchors are now beginning to appear. TREM2 has been reported to improve microglial function and synaptic development in ASD-relevant settings through p38 MAPK-related signaling (43). Other studies suggest that TREM2 deficiency can contribute to excitation-inhibition imbalance and ASD-like behavioral phenotypes through Kv1.3-dependent mechanisms (44), and that TREM2 downregulation may disrupt microglial synaptic pruning through RA/RARα-related signaling (45). These studies do not define one dominant ASD pathway, but they strengthen the case that receptor-linked metabolic support and phagocytic competence are directly relevant to neurodevelopmental phenotypes.

### 5.2 Lipid storage, lysosomal load, and functional competence

Lipid storage is another informative area. Lipid-droplet-accumulating microglia were described in aging as a dysfunctional, pro-inflammatory phenotype associated with impaired phagocytosis and altered lipid metabolism (46). This literature cannot be mapped directly onto ASD, because age, tissue environment, and disease substrate are very different. Nevertheless, it provides a useful interpretive principle: lipid accumulation may indicate impaired cellular processing capacity rather than simply increased activation.

This principle is relevant to ASD because recent human and experimental studies repeatedly point to lysosomal, phagocytic, and lipid-handling pathways in glial compartments (11, 24). The key question is therefore not whether an ASD-associated microglial signature resembles a known lipid-storage phenotype at the transcript level, but whether it is associated with altered engulfment capacity, lysosomal throughput, or synapse-related substrate handling. This distinction is important because it separates descriptive molecular overlap from functional interpretation.

Lysosomal burden deserves particular attention in this context. Increased expression of lysosomal genes can reflect active degradation, increased substrate load, incomplete clearance, or some combination of these processes. Without accompanying functional assays, lysosomal signatures in ASD should not be assumed to indicate either enhanced or impaired microglial competence. This is one reason why immunometabolic interpretation is most informative when transcriptomic or proteomic readouts are paired with direct assessments of cargo processing and synapse-related function.

### 5.3 Mitochondrial function and energetic constraints

Mitochondrial and glycolytic regulation provide a related perspective on microglial competence. Recent work outside ASD has shown that metabolic coupling can directly influence phagocytic ability. For example, disruption of the TSPO-hexokinase 2 axis was reported to impair respiratory-glycolytic coupling and phagocytosis in microglia (47). Such studies are important because they move beyond descriptive inflammatory labels and show that metabolic nodes can alter what microglia are functionally able to do.

This point is particularly relevant to ASD because many proposed microglial effects concern synapse-facing functions rather than cytokine output alone. Process motility, surveillance, engulfment, and lysosomal degradation all impose energetic costs. Recent *in vivo* work has further shown that mitochondria are largely absent from distal microglial processes engaged in surveillance, chemotaxis, and phagocytic engulfment, emphasizing that the subcellular deployment of metabolic machinery is itself spatially constrained (48). Although these findings do not establish an ASD-specific mechanism, they make clear that metabolic state can shape the capacity of microglia to execute developmental and synaptic functions.

For ASD interpretation, the major lesson is methodological as well as biological. Metabolic readouts are most informative when linked to direct assays of lysosomal load, engulfment, process behavior, or synapse remodeling. Without that functional linkage, the biological meaning of metabolic signatures remains underdetermined.

### 5.4 Bioactive lipid mediators and inflammatory-metabolic coupling

Bioactive lipid mediators further illustrate how inflammatory and metabolic pathways converge. In Alzheimer’s disease models, prostaglandin E2 signaling through EP2 suppressed beneficial microglial functions, indicating that inflammatory lipid mediators can act as functional brakes rather than merely as markers of activation (49). This point is conceptually useful for ASD because stronger inflammatory tone does not automatically imply stronger or more effective synapse remodeling. In some settings, inflammatory lipid signaling may instead accompany impaired microglial competence.

Direct evidence for prostaglandin-centered microglial mechanisms in ASD remains limited. At present, these pathways are better viewed as candidate modulators of microglial capacity than as established ASD mechanisms. Their main value here is that they reinforce a broader interpretive point: lipid mediators can reshape what microglia are able to do, not simply how inflammatory they appear in bulk molecular summaries.

### 5.5 Developmental relevance of immunometabolism in ASD

A major concern in this area is that much of the detailed immunometabolism literature comes from aging and neurodegeneration, whereas ASD is fundamentally a neurodevelopmental condition. That concern is valid, but it does not make metabolic framing irrelevant to ASD. Developmental microglia undergo age-dependent metabolic transitions, and recent synthesis work has specifically highlighted the relevance of these transitions to neurodevelopmental disorders, including ASD (50).

Experimental ASD models add support to this perspective. In the Neuroligin-4 knockout model, microglia showed sex-dependent metabolic and functional abnormalities (22). Human microglia carrying ASD-risk perturbations also displayed altered endocytosis and pruning capacity in CRISPRi-based studies (24). Together with the TREM2-related studies noted above, these findings indicate that immunometabolism is not merely an analogy borrowed from neurodegeneration; it is increasingly relevant to direct ASD-related microglial phenotypes.

Overall, the current literature supports a balanced conclusion. Immunometabolic pathways are likely to be important regulators of microglial behavior in ASD-relevant contexts, especially those involving lipid handling, receptor-linked metabolic support, lysosomal function, and synapse-related substrate processing. However, ASD evidence is still developing, and it remains premature to treat one metabolic profile as a universal signature of ASD microglia. Current evidence indicates that immunometabolism provides a useful mechanistic framework for interpreting ASD-related microglial variation, but only when tied to developmental context and direct functional readouts.

## 6 Microglia-synapse interfaces in ASD

Microglial effects on neural circuits are most directly expressed at the synapse. Yet in the ASD literature, conclusions about synaptic pruning are often inferred from immune or phagocytic signatures rather than from synapse-facing measurements. This is a major interpretive problem, because excessive, insufficient, and mistargeted pruning can all produce glia-associated molecular changes but have very different implications for circuit development. A more precise review of the field therefore needs to focus on pathways that operate directly at the microglia-synapse interface.

### 6.1 Complement-linked tagging

The complement cascade remains the best-established mechanistic entry point. Classical complement components can localize to developing synapses and help mark them for microglia-dependent elimination during critical windows of circuit refinement (6, 51). This literature is central to ASD interpretation because it shows that the biologically informative event is not simply elevated complement gene expression, but synapse-associated tag deposition coupled to microglial recognition and engulfment.

In ASD, complement-related abnormalities have been reported in postmortem brain tissue and in specific niches such as the subventricular zone (20), but the direction and functional meaning of these abnormalities still need to be resolved at the synaptic level rather than inferred from transcript abundance alone. Complement components can arise from multiple cellular sources, and their biological significance depends on localization, timing, and coupling to actual remodeling events.

Experimental ASD-relevant systems now provide more direct evidence. In SCN2A-deficient mice and human cerebral organoids, microglia were reported to drive excessive synaptic pruning during development, linking ASD-relevant neuronal perturbation to an over-pruning phenotype (35). These studies are important because they move the field from general inflammatory association toward defined synapse-remodeling mechanisms. At the same time, they also illustrate a broader point: even when microglia are functionally involved, the dominant pruning phenotype may depend on developmental timing, neuronal substrate, and circuit class. For this reason, ASD-related complement biology is best interpreted as a candidate mechanism that must be validated within specific synaptic and developmental contexts.

### 6.2 Phosphatidylserine-dependent recognition

Complement is not the only way synapses become eligible for elimination. Phosphatidylserine exposure on neuronal elements provides another major route through which microglia recognize material for clearance. Developmental work has shown that local phosphatidylserine externalization can function as a neuronal signal for microglial synapse elimination and that this process is linked to receptor systems including TREM2-related recognition (52).

This pathway is especially relevant to ASD because it emphasizes that synapse elimination is not simply determined by inflammatory tone. Instead, it depends on the presence of specific eat-me signals at defined synaptic sites. This makes phosphatidylserine-related pathways useful mechanistic candidates when interpreting ASD-associated microglial changes, particularly in settings where pruning is proposed but direct evidence remains limited.

### 6.3 Synapse-class specificity and MERTK-related pathways

Related work has also demonstrated that microglial pruning can be synapse-class specific. Microglial MERTK-dependent elimination of phosphatidylserine-exposing inhibitory post-synapses has been shown *in vivo*, indicating that synapse type matters for interpreting pruning mechanisms (53). This point is highly relevant for ASD, where excitation-inhibition balance is a recurring theme across genetics, circuit physiology, and postmortem transcriptomics. If microglia contribute to ASD-related synaptic dysfunction, their effects may not be uniform across excitatory and inhibitory compartments.

For this reason, pruning hypotheses in ASD are strongest when they specify the relevant synapse class, developmental window, and circuit context rather than invoking pruning as a single undifferentiated process. This is another area in which broad activation terminology is inadequate, because it does not indicate which synapses are being affected, when, or through which recognition system.

### 6.4 Anti-engulfment checkpoints

A second important consideration is that pruning is not determined only by pro-engulfment signals. Anti-engulfment checkpoints also constrain when and where microglia remove synaptic material. One well-characterized example is CD47-SIRPα signaling, which protects synapses from excessive developmental pruning (54). This literature is conceptually important for ASD because it helps explain why increased phagocytic or lysosomal gene expression does not automatically mean increased pruning. Depending on the balance between synaptic tags and synaptic protection signals, similar microglial transcriptional states could be associated with excessive, insufficient, or misdirected elimination.

Accordingly, studies that propose microglia-dependent synaptic changes in ASD are more informative when they assess both tags and brakes. Complement deposition, phosphatidylserine exposure, checkpoint signaling, and synapse-class-resolved engulfment should ideally be interpreted together rather than separately. This approach is more likely to distinguish excessive from insufficient elimination than descriptive state labels alone.

### 6.5 Remodeling beyond whole-synapse engulfment

High-resolution imaging studies have further shown that microglia do not remodel synapses only through complete engulfment. They can also perform presynaptic trogocytosis and induce spine head filopodia, thereby modifying synaptic connectivity through partial remodeling events (7). This observation broadens the range of plausible ASD-related phenotypes. Some contexts may involve overt synapse loss, whereas others may involve altered remodeling, atypical contact dynamics, or changes in synapse stabilization without large-scale engulfment.

Current evidence does not support collapsing all microglial influence in ASD into a single pruning model. Studies aimed at linking microglia to ASD-relevant synaptic outcomes are more informative when they incorporate assays capable of distinguishing complete engulfment from partial remodeling.

### 6.6 Astrocyte-microglia crosstalk at the synapse

Astrocytes add another layer of complexity. They can directly engulf synapses through MEGF10- and MERTK-dependent pathways (8), and microglia can induce astrocytic states that alter inflammatory tone and synaptogenic support (9). Recent ASD-oriented synthesis work has further emphasized the relevance of non-neuronal interactions and astrocyte-microglia crosstalk for synaptic and circuit phenotypes (55).

These findings do not reduce the importance of microglia. Rather, they show that microglia-synapse interactions in ASD are best understood within a broader glial network. This is particularly important because some synaptic outcomes attributed to microglia may in practice reflect distributed glial responses rather than a purely microglia-intrinsic process.

### 6.7 Informative readouts at the microglia-synapse interface

Taken together, the current literature supports a more disciplined way of discussing synaptic pruning in ASD. The key question is not simply whether microglia appear reactive, but whether defined synapse-facing mechanisms are engaged in a manner consistent with a specific developmental and circuit phenotype. In practice, this means that complement deposition, phosphatidylserine exposure, anti-engulfment checkpoints, lysosomal localization of synaptic cargo, trogocytosis, and synapse-class specificity are more informative than broad inflammatory descriptors alone.

This framework also helps clarify how different pruning hypotheses should be evaluated. Excessive, insufficient, and mistargeted synaptic remodeling are not interchangeable possibilities, and they should not be inferred from the same kinds of evidence. The strongest ASD studies in this area are likely to be those that integrate molecular signatures with direct synapse-level readouts and with careful attention to timing, region, and cell-type context.

## 7 Developmental timing, sex, and biological context

One reason why ASD microglia findings can appear inconsistent is that microglial biology is strongly shaped by developmental stage, sex, anatomical niche, and systemic environment. These factors do not merely add noise around a central signal. They influence baseline microglial state, response thresholds, metabolic demands, and effector outputs. As a result, the same nominal immune- or glia-associated signature may have different meanings depending on when and where it is observed.

### 7.1 Developmental timing

Developmental timing is especially important. Microglia undergo stage-specific maturation programs across prenatal, early postnatal, adolescent, and adult periods, with substantial shifts in gene regulation and function over time (56). Because ASD is a neurodevelopmental condition, this temporal dimension matters directly for interpretation. Many human ASD datasets derive from adolescent or adult postmortem tissue, whereas mechanisms relevant to synaptic pruning and circuit assembly are likely to operate earlier. Immune- and glia-associated alterations observed later in life may therefore reflect ongoing pathology, secondary adaptation, or residual traces of earlier developmental disturbance rather than the primary initiating event.

This temporal issue is one reason why mechanistic extrapolation from adult human tissue requires caution. It also explains why developmentally grounded comparison with experimental systems and developmental microglia references remains essential when interpreting human ASD data.

### 7.2 Sex as an interpretive dimension

Sex is another major modifier. Experimental studies have shown sex-dependent differences in microglial maturation and immune responsiveness (57), and recent analyses suggest broader sex-dependent divergence in human microglial developmental trajectories (58). These observations are directly relevant to ASD given its marked sex bias.

In ASD-relevant models, microglial phenotypes can also be sex conditional. The Neuroligin-4 knockout model showed sex-specific metabolic and functional changes in microglia (22), and Cntnap2-related work linked sexually dimorphic microglial activity and synaptic phagocytosis to circuit abnormalities (59). These findings suggest that at least some discrepancies across ASD studies may reflect genuine sex-dependent biology rather than lack of reproducibility alone. In interpreting the literature, sex should therefore be treated as an interpretive dimension rather than as a demographic detail.

### 7.3 Anatomical niche and neurovascular context

Microglia are also shaped by local tissue context. They interact closely with the neurovascular unit and can influence blood-brain barrier properties, vascular signaling, and local inflammatory tone (60, 61). During development, pericyte-microglia interactions can help maintain microglial homeostasis and influence progenitor dynamics (62).

These relationships matter for ASD because different brain regions vary in developmental history, cellular composition, and niche structure. Cortex, amygdala, subventricular zone, and cerebellum do not provide equivalent microglial contexts. Accordingly, findings from one region should not automatically be generalized to another without attention to local developmental and anatomical conditions.

### 7.4 Systemic and environmental influences

Systemic factors are relevant as well. The microbiota influences microglial maturation (63), maternal immune activation alters fetal and neonatal microglial phenotypes (64, 65), and maternal metabolic inflammation has been proposed to prime microglial responses in ASD-relevant developmental contexts (66, 67). Gene-by-environment interactions further support this view. In experimental systems, Cntnap2 deficiency can interact with maternal immune activation in shaping ASD-relevant offspring phenotypes, while maternal allergic asthma has been associated with prenatal neuroinflammation and persistent microglia-related molecular alterations in offspring (68–70).

These observations do not imply that systemic or environmental factors explain all ASD-related microglial findings. Rather, they indicate that microglial phenotypes are partially niche determined and may be influenced by developmental exposures outside the brain parenchyma itself. This broader context is especially important when comparing human postmortem studies, animal models, and *in vivo* biomarkers, because each reflects different mixtures of central and systemic biology.

### 7.5 Practical implications for interpreting ASD studies

These contextual factors have direct practical implications. Brain region matters because microglia in different regions experience different substrates and developmental histories (19, 20). Donor structure matters because single-cell and spatial datasets can be distorted by pseudo-replication if donor-level variability is not modeled appropriately (71, 72). Clinical composition also matters, including epilepsy, psychiatric comorbidities, medication exposure, intellectual disability, and cause-of-death variables, all of which may affect neuroimmune and metabolic signatures independently of ASD diagnosis itself.

These considerations also help explain why postmortem and *in vivo* neuroimmune studies do not always agree. Postmortem data capture tissue-level molecular and histological states shaped by region, developmental history, agonal factors, RNA quality, and treatment exposure. By contrast, TSPO PET measures a living glia-associated signal influenced by ligand characteristics, genotype, mitochondrial biology, and clinical subgroup structure (16, 17, 29, 30). Lack of one-to-one correspondence between these modalities should therefore not be taken as simple contradiction. They capture related but distinct layers of biology.

Overall, developmental timing, sex, anatomical niche, and systemic context should be treated as central interpretive dimensions in ASD microglia research rather than as secondary qualifiers. Consideration of these factors helps explain why the literature shows partial convergence without supporting a single uniform ASD microglial phenotype.

## 8 Interpreting microglial findings in ASD

**Figure 3:**
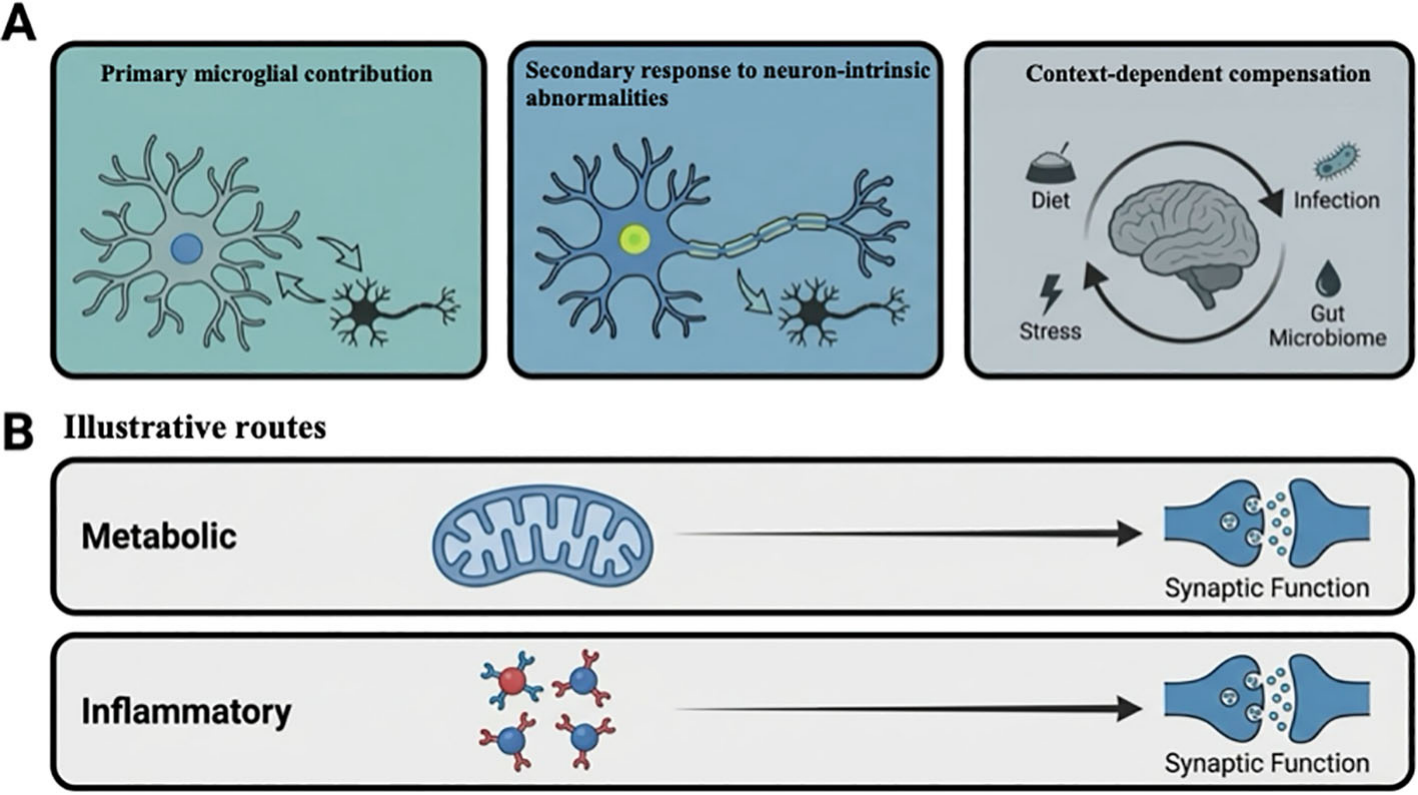
Conceptual models for interpreting microglial findings in ASD. **(A)** The upper panel summarizes three non-exclusive interpretive models discussed in the literature: primary microglial contribution, secondary response to neuron-intrinsic abnormalities, and context-dependent compensation. These models may coexist across developmental stages, brain regions, and ASD subgroups. **(B)** The lower panel illustrates two example routes, metabolic and inflammatory, through which microglia-related processes may influence synaptic function.

Current evidence supports several non-exclusive ways of interpreting microglial findings in ASD. In some studies, microglial alterations are discussed as potentially contributing directly to synaptic remodeling or circuit maturation. In others, they are interpreted as responses to neuron-intrinsic developmental abnormalities or broader tissue-level disturbance. A further possibility is that some signatures reflect partial or context-dependent compensation. These possibilities are not mutually exclusive, and their relative relevance likely varies across developmental stages, brain regions, and ASD subgroups. **Figure 3** summarizes three interpretive models discussed in the literature: primary microglial contribution, secondary responses to neuron-intrinsic abnormalities, and context-dependent compensation.

A microglia-first interpretation is most strongly supported when direct perturbation of microglial pathways precedes and alters synaptic or behavioral phenotypes in ASD-relevant systems. This type of evidence is now beginning to emerge. For example, recent studies implicating TREM2-related signaling in ASD-relevant synaptic development and behavior suggest that at least some neurodevelopmental phenotypes may be modified through defined microglial mechanisms rather than through general inflammation alone (43–45). CRISPRi-based studies in human microglia also support this direction by showing that ASD risk-gene perturbation can directly alter endocytosis and pruning-related functions (24).

A neuron-first interpretation remains equally plausible in many ASD contexts. A large proportion of high-confidence ASD risk genes act primarily in neurons, synaptic scaffolds, chromatin regulators, or translational control pathways rather than in microglia. In these cases, altered microglial states may reflect responses to abnormal neuronal activity, altered synaptic turnover, or atypical circuit maturation. This interpretation is consistent with the fact that many human ASD transcriptomic studies identify parallel neuronal and glial abnormalities rather than isolated microglial signatures (3) (4, 10).

A compensation-oriented interpretation is especially relevant when immune- and metabolism-related signatures do not map cleanly onto a single functional direction. Increased lysosomal, phagocytic, or lipid-handling pathways may in some settings reflect productive microglial engagement, but in others they may reflect substrate overload, incomplete clearance, or energetically strained adaptation. This reading is particularly useful for interpreting immunometabolic findings because it avoids assuming that transcriptional evidence of engagement automatically implies effective function.

These three interpretations help explain why similar molecular signatures can be associated with different proposed outcomes across studies. They also highlight a major gap in the current field: most human evidence is still better at identifying associated biology than at distinguishing primary pathogenic mechanisms from secondary response or compensation. Progress will therefore depend on designs that connect human cell-resolved findings to perturbation-capable systems and to synapse-facing functional readouts.

### 8.1 Implications for biomarkers

Interest in microglia as biomarkers in ASD has increased alongside the broader literature on neuroimmune involvement. However, current evidence does not support a single validated microglia-specific biomarker for ASD. TSPO PET is informative as a living glia-associated measure, but it is not microglia specific, and published ASD findings remain heterogeneous (27–30). Postmortem molecular signatures are also valuable, but they do not define ongoing cellular function in living individuals. Peripheral immune measures may provide additional context, yet their relationship to CNS-resident microglial biology is indirect.

This does not mean biomarker work is uninformative. Rather, it suggests that future biomarker strategies will likely need to be subgroup aware and multimodal. Biomarkers are more likely to be informative when they are linked to a specific mechanistic hypothesis, such as altered complement-related tagging, impaired phagocytic competence, or metabolism-constrained microglial function, than when they are framed as general evidence of neuroinflammation.

### 8.2 Implications for therapy

The same caution applies to therapy. Current literature does not justify broad suppression or activation of microglia as a generalized ASD treatment strategy. If microglia contribute to ASD-related phenotypes, that contribution is likely to depend on mechanism, developmental timing, sex, and subgroup structure. Therapeutic relevance is therefore most plausible for pathway-defined targets, including receptor-linked pruning mechanisms, metabolic support pathways, or specific glia-synapse interactions, rather than for non-specific anti-inflammatory approaches (73–75).

For neurodevelopmental disorders, timing is especially important. A pathway that shapes early synaptic refinement may not remain equally modifiable later in life, and later intervention may affect compensation rather than the original developmental process. Therapeutic interpretation in ASD must therefore remain tied to developmental context and to evidence that a given pathway is both mechanistically relevant and temporally actionable (76, 77).

## 9 Remaining limitations and priorities

**Figure 4:**
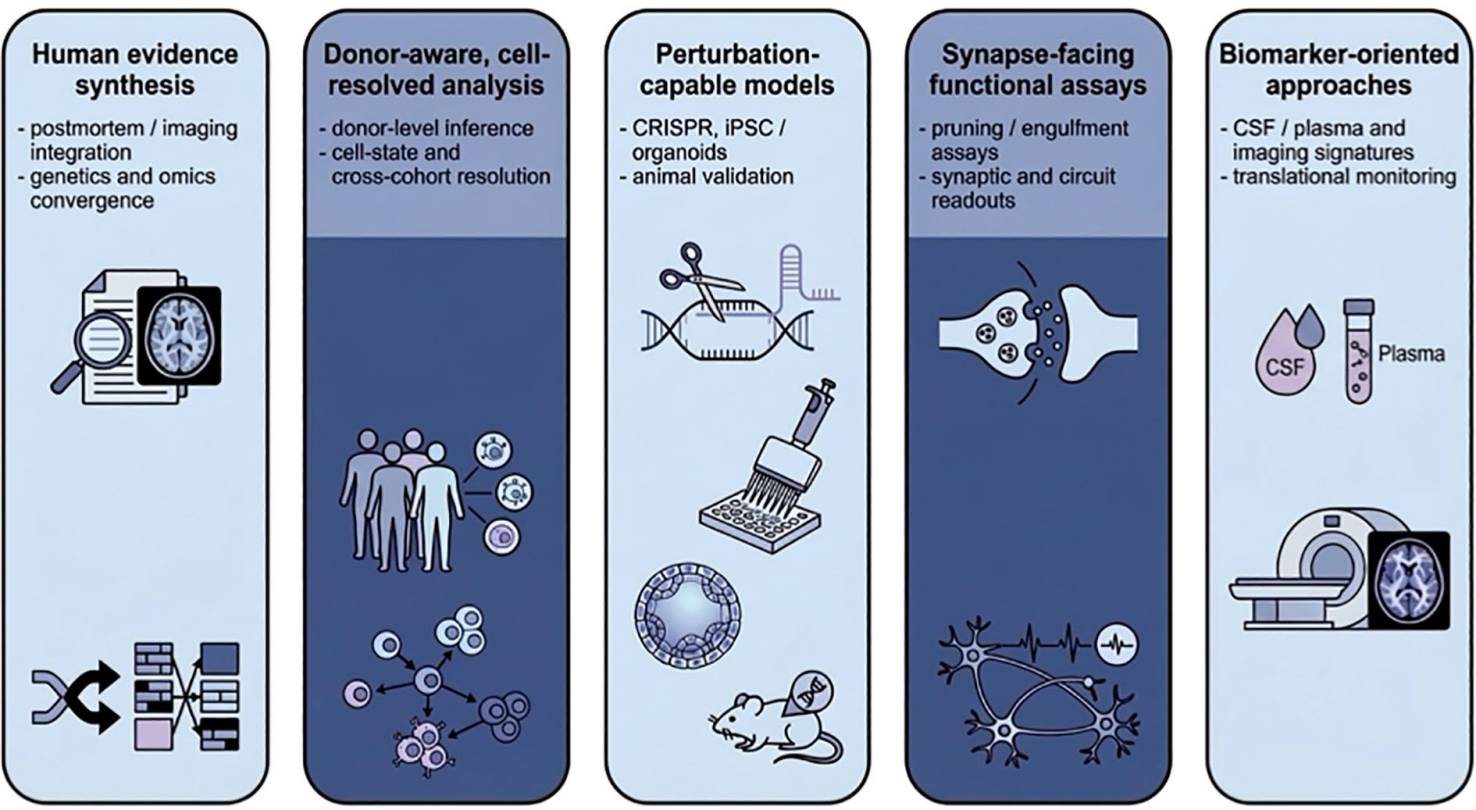
Methodological and research priorities in ASD microglia studies. The figure summarizes several methodological priorities discussed in this Review, including human evidence synthesis, donor-aware cell-resolved analysis, perturbation-capable models, synapse-facing functional assays, and biomarker-oriented approaches. Rather than depicting a single linear workflow, the figure highlights study-design considerations that may help clarify when microglial alterations in ASD reflect primary contributions, secondary responses, or context-dependent processes.

Despite substantial progress, several limitations continue to constrain interpretation of microglial findings in ASD. These limitations help define the main areas in which stronger evidence is still needed. **Figure 4** summarizes methodological and research priorities that may help strengthen causal and interpretive inference in ASD microglia studies.

### 9.1 Human evidence remains largely associative

The first limitation is that most human evidence remains associative. Postmortem histology and molecular profiling provide important biological detail but limited temporal inference. Bulk transcriptomic studies have identified convergent immune- and glia-related patterns in ASD cortex, but they cannot fully separate cell-intrinsic shifts from cell-composition changes (3, 4). Even single-cell and spatially resolved datasets, while substantially more informative, are still typically cross-sectional and often based on modest and heterogeneous donor collections (10). As a result, current human studies are better at defining recurrent microglia-relevant patterns than at determining whether those patterns are causal, secondary, adaptive, or mixed.

This limitation is especially important in ASD because developmental timing is central to interpretation. Many mechanisms of interest, particularly those related to synaptic refinement, are likely to operate during restricted developmental windows, whereas much of the available human material comes from adolescent or adult brain tissue. Consequently, later-stage molecular signatures may reflect persistent pathology, residual traces of earlier developmental perturbation, or later compensatory responses rather than the initiating event itself (76, 77).

### 9.2 Cellular specificity and inferential limits

A second limitation concerns cellular specificity. Many commonly used readouts are glia-associated rather than microglia-specific. This issue applies particularly to TSPO PET, some inflammatory markers, and some transcriptomic modules that overlap with astrocytic or infiltrating myeloid programs. Stronger inference therefore requires continued use of cell-type-resolved and spatially localized approaches, together with precise language about what each modality can and cannot support (78) (79).

This point is not merely semantic. Overly broad labels can make distinct biological scenarios appear more similar than they really are. For example, increased glia-associated transcripts could reflect changes in microglia, astrocytes, vascular-associated cells, cellular proportions, or several of these together. Review and primary-study interpretations should therefore continue to distinguish carefully among tissue-level, cell-type-level, and mechanism-level claims, and should use “activation” terminology cautiously unless it is explicitly defined and directly justified by the data (80, 81).

### 9.3 Developmental and regional alignment

A third limitation is developmental and anatomical alignment. Much of the mechanistic interest in microglia relates to early synaptic refinement, yet much of the available human material comes from adolescent or adult tissue. Likewise, different regions are often discussed together even though cortex, amygdala, subventricular zone, and cerebellum differ substantially in developmental trajectory and local niche structure. Future studies will be more informative when developmental stage, anatomical sampling, and proposed mechanism are aligned more explicitly (76).

This issue is especially relevant for pruning-related hypotheses. Synapse elimination is strongly influenced by developmental timing, synapse class, and circuit context. A mechanistic claim drawn from one region or age range should therefore not be generalized too broadly without evidence that the same biology is present in the relevant ASD context (75, 77).

### 9.4 Subgroup structure, metadata quality, and donor-aware analysis

A fourth limitation is subgroup structure. Sex, epilepsy, intellectual disability, psychiatric comorbidity, medication exposure, and systemic immune or metabolic status all have the potential to alter neuroimmune and glial biology independently of ASD diagnosis itself. Because ASD is clinically heterogeneous, subgroup-aware study design is not optional. It is essential if the field is to move beyond inconsistent average case-control findings.

Metadata quality also matters. Postmortem interval, RNA quality, tissue pH, cause of death, agonal state, seizure history, and psychotropic treatment exposure can all affect molecular readouts. These issues are particularly consequential in neuroimmune studies because microglial and astrocytic signatures can be highly responsive to tissue condition and physiological stress.

At the analytic level, donor-aware modeling is equally important. Single-cell and spatial studies can be distorted by pseudo-replication if cells are treated as independent biological replicates, and benchmarking work has emphasized the importance of accounting for between-donor variation and using pseudobulk or related donor-aware strategies when appropriate (71, 72). For ASD microglia studies, this is not a technical detail but a core interpretive safeguard.

### 9.5 From molecular signatures to functional readouts

A fifth limitation concerns functional interpretation. Increased expression of lysosomal, complement, phagocytic, or lipid-handling genes does not by itself establish whether synapse remodeling is excessive, insufficient, mistargeted, or functionally ineffective. This remains one of the most important interpretive challenges in the field. Future work will be strongest when descriptive molecular signatures are paired with direct readouts such as synaptic tag localization, phosphatidylserine exposure, checkpoint signaling, lysosomal cargo quantification, engulfment metrics, trogocytosis, and synapse-class-specific outcomes (75).

This priority applies equally to human-referenced model systems and to *in vitro* perturbation platforms. Functional assays are especially valuable when they can distinguish whether a given molecular program reflects effective execution of a microglial task, incomplete adaptation to increased substrate burden, or loss of competence despite apparent activation-related transcriptional change. Recent perturbation-capable systems in SCN2A-deficient mouse–organoid models and CRISPRi-based human microglia platforms illustrate how molecular signatures can be linked more directly to pruning, endocytosis, and lysosomal phenotypes (24, 35).

### 9.6 Model systems and links to genetics

Model systems remain indispensable because they allow perturbation-based inference, but they also need to be interpreted with care. ASD is genetically and biologically heterogeneous, and not every model-derived microglial phenotype should be generalized across the spectrum. The most informative models are therefore likely to be those that link defined genetic or environmental perturbations to mechanism-specific microglial functions and can be evaluated against human cell-resolved datasets rather than in isolation.

This is one reason why recent perturbation-capable systems are so useful. Human microglia CRISPRi platforms, organoid-based co-culture systems, and genetically defined mouse models can connect ASD risk architecture to defined microglial outcomes such as endocytosis, synaptic pruning, lysosomal processing, and receptor-linked signaling (24, 35). When interpreted together with human tissue data, these systems provide a more rigorous route from association to mechanism.

### 9.7 Biomarkers and translational priorities

Translation is an important but still developing dimension of the field. Current evidence does not yet support a clinically actionable microglial biomarker for ASD, and it does not justify indiscriminate neuroimmune intervention. More broadly, clinical evidence for immunoregulatory or anti-inflammatory treatment strategies in ASD remains limited and heterogeneous, arguing against non-specific inflammation-based therapeutic framing at present (73, 74). Therapeutic relevance is therefore more plausibly linked to pathway-defined targets, including receptor-linked pruning mechanisms, metabolic support pathways, or specific glia–synapse interactions, rather than to generalized suppression or activation of microglia (75).

For neurodevelopmental disorders, timing is especially important. A pathway that shapes early synaptic refinement may not remain equally modifiable later in life, and later intervention may affect compensation rather than the original developmental process. Therapeutic interpretation in ASD must therefore remain tied to developmental context and to evidence that a given pathway is both mechanistically relevant and temporally actionable (76, 77). More broadly, future studies are likely to be most informative when they combine donor-aware single-cell and spatial analysis, explicit developmental alignment between human and experimental systems, subgroup-aware design, and synapse-facing functional assays. Integrative studies that connect human genetics, cell-resolved molecular profiling, perturbation-capable models, and biomarker development are especially likely to clarify when microglia act as drivers, responders, or compensatory participants in ASD-related biology.

### 9.8 A practical priority for future reviews and primary studies

In parallel with new experiments, the field also needs continued improvement in how evidence is described. Broad activation terminology should be used cautiously unless it is explicitly defined and directly justified by the data. A vocabulary centered on cell context, mechanism, developmental timing, and inferential scope will improve both clarity and comparability across studies. This is particularly important in ASD, where partial convergence across modalities coexists with substantial biological heterogeneity (80, 81).

## 10 Conclusions

The literature reviewed here supports a measured but increasingly informative view of microglial involvement in ASD. Microglia are unlikely to be captured by a single ASD-wide phenotype, and broad activation terminology is often inadequate for interpreting the diversity of human and experimental evidence. Instead, current studies point to context-dependent microglial heterogeneity, immunometabolic regulation, and synapse-related pathways whose significance varies with developmental timing, sex, brain region, and local tissue environment.

Human postmortem and transcriptomic studies support recurrent immune- and glia-associated alterations in at least a subset of ASD brains (3, 4, 11, 12). Cell-resolved and spatial approaches have strengthened this picture by showing that these alterations occur within a broader multicellular context that includes neuronal, astrocytic, oligodendroglial, and vascular-associated changes (19–21).

Experimental studies and perturbation-capable systems further suggest that pathways involving complement, phosphatidylserine recognition, anti-engulfment checkpoints, lipid handling, lysosomal processing, and receptor-linked metabolic support may influence microglial contributions to synaptic remodeling in ASD-relevant settings (24, 35, 43, 52, 53).

At the same time, substantial uncertainty remains. Current evidence does not yet determine whether the dominant microglial contribution in ASD is best described as excessive, insufficient, or mistargeted synaptic remodeling across all contexts, nor does it fully resolve whether microglial changes are primary, secondary, or partially compensatory. These questions remain open because most human evidence is still cross-sectional and because many commonly used readouts are better at identifying associated biology than at establishing functional direction. The need for donor-aware inference in single-cell studies and for developmentally aligned interpretation remains especially important in this context (50, 71, 72).

Overall, the literature supports viewing microglia as context-dependent participants in ASD-related brain biology rather than as a single explanatory mechanism. Further progress will depend on integrating donor-aware human datasets, developmentally aligned model systems, and mechanism-linked functional readouts in ways that improve temporal resolution, cellular specificity, and interpretability.

## Supplementary material

Supplementary Table 1 Detailed evidence summary for primary ASD microglia-relevant studies. Expanded version of **Table 1**, including modality/readouts, a one-line summary of ASD-relevant microglia findings, and major confounders/notes for each primary study.

Supplementary Table 2 Modality-specific confounders and interpretation safeguards. Common confounders, recommended safeguards, and interpretation notes across postmortem, transcriptomic, imaging, genetic, and experimental modalities relevant to ASD microglia research.

Supplementary Table 3 Microglia-related states, pathways, and functional themes discussed in ASD literature. Representative microglia-related concepts recurrent across ASD studies, including states, pathways, functional themes, and key caveats for cross-study interpretation.
