# Supplementary material for: Microglia in autism spectrum disorder: heterogeneity, immunometabolism, and synapse-related pathways
Source: Front Immunol. 2026 Apr 28;17:1783755. doi: 10.3389/fimmu.2026.1783755 (PMC13160863; doi:10.3389/fimmu.2026.1783755)
Supplement: Supplementary Table 1 — Detailed evidence summary for primary ASD microglia-relevant studies. Expanded version of Table 1, including modality/readouts, a one-line summary of ASD-relevant microglia findings, and major confounders/notes for each primary study. [file DataSheet1.pdf]

Supplementary Material

Contents

- Supplementary Methods
- Text S1. Targeted search strategy and study prioritization
- Supplementary Tables
- Table S1. Detailed evidence summary for primary ASD microglia-relevant studies
- Table S2. Modality-specific confounders and interpretation safeguards
- Table S3. Microglia-related states, pathways, and functional themes discussed in ASD literature
- Supplementary Figures
  Figure S1. Literature identification and evidence organization workflow

Supplementary Methods

Text S1. Targeted search strategy and study prioritization

We conducted targeted searches in PubMed and Google Scholar from database inception to January 2026. Search strings used combinations of ASD terms with microglia-related and topic-specific blocks, for example:

(i) ("autism" OR "ASD") AND microglia AND ("single-cell" OR "single nucleus" OR "spatial" OR "postmortem");

(ii) ("autism" OR "ASD") AND microglia AND ("synaptic pruning" OR complement OR engulfment OR trogocytosis);

(iii) ("autism" OR "ASD") AND microglia AND ("immunometabolism" OR lipid OR mitochondria OR glycolysis OR "oxidative phosphorylation" OR lysosome).

We prioritized (a) human postmortem brain studies and in vivo imaging with clearly reported cohort metadata; (b) cell-type-resolved omics and spatial data enabling microglia-specific interpretation; and (c) ASD-relevant model systems with perturbation-based evidence linking microglial biology to synapse-related functional readouts. Additional references were identified via citation chaining (reference lists and "cited-by" screening) from landmark studies and recent reviews. A detailed study-level evidence summary (readouts and major confounders) is provided in Table S1. Modality-specific interpretation safeguards and confounder considerations are summarized in Table S2.

Supplementary Tables

Table S1. Detailed evidence summary for primary ASD microglia-relevant studies

Purpose: Expanded version of Table 1, including modality/readouts, a one-line summary of ASD-relevant microglia findings, and major confounders/notes for each primary study.

Evidence Type	Study (Author, Year)	System / Cohort / Model	N (ASD / Ctrl or per Group)	Sex (M/F)	Age / Stage	Brain Region / Tissue	Microglia-related Readout(s)	Key ASD-Relevant Microglia Point	Major Confound(s) / Notes
**Human Postmortem Transcriptomics**	**Voineagu, 2011**	Postmortem human brain	36 donors (19 ASD / 17 Ctrl); 58 samples	26M / 10F	Range: 2–56 years	STG (BA41/42), PFC (BA9), Cerebellum	Co-expression modules (asdM16); A2BP1/FOX1 splicing	Identified asdM16 module enriched in glial markers linked to transcriptional dysregulation	PMI, RIN, age, and medication history
**Human Postmortem Transcriptomics**	**Gupta, 2014**	Postmortem human brain	72 donors (32 ASD / 40 Ctrl); 104 samples	Sex matched; exact M/F NR	Median: ASD 20 years / Ctrl 17 years	BA10 (frontal pole), BA44, BA19	Co-expression (mod5); type I IFN response genes	Pinpointed asdM16 to innate immunity and M2-state microglial response	PMI, RIN, and brain bank source
**Human Postmortem Transcriptomics**	**Velmeshev, 2019**	Human postmortem snRNA-seq	31 donors (15 ASD / 16 Ctrl); 41 samples	ASD: 13M / 2F; Ctrl: exact M/F NR (matched)	Range: 4–22 years	PFC (BA10) and ACC (BA24)	Activation markers; developmental TFs (AHI1)	ASD microglia are enriched for activation genes and dysregulated developmental TFs	Clinical severity (ADI-R scores) correlations
**Human Postmortem Transcriptomics**	**Wamsley, 2024**	Human postmortem snRNA/ATAC	63 donors (33 ASD / 30 Ctrl)	Sex matched; exact M/F NR	Range: 2–60 years (Mean: 20 years)	Frontal cortex (BA9, BA4/6)	Glia-reactivity GRNs (IRF8); MG2 state proportion	Identified reactive MG2 cluster significantly increased in ASD frontal cortex	Included 5 (dup)15q11-13 monogenic cases
**Human In Vivo Imaging**	**Suzuki, 2013**	Human 11C-PK11195 PET	40 participants (20 ASD / 20 Ctrl)	40M / 0F (100% Male	Range: 18–31 years	Cerebellum, midbrain, pons, ACC,	11C-PK11195 Binding Potential (BP)	First in vivo evidence of augmented microglial activation in	IQ matched (high and low IQ subsets)

				)		OFC		multiple brain regions	
**Human In Vivo Imaging**	**Zürcher, 2021**	Human [11C]PBR28 MR-PET	33 participants (15 ASD / 18 Ctrl)	33M / 0F (100 % Male)	ASD: 24.1 ± 5.5 years; Ctrl: 25.5 ± 5.8 years	Insula, Putamen, PCC, OFC, STG	TSPO regional distribution (SUVR)	Reported lower regional TSPO expression, suggesting a distinct neuroimmune profile in adult males	TSPO genotype (Ala147Thr) correction
**Human In Vivo Imaging**	**Tseng, 2024**	Human [11C]PBR28 PET-MRI pilot	22 participants (12 ASD / 10 Ctrl)	0M / 22F (100 % Female)	ASD: 25.33 ± 5.88 years; Ctrl: 25.70 ± 4.62 years	Midcingulate cortex (MCC), CC splenium	[11C]PBR28 Binding Potential (BP)	Pilot study in females showing regional TSPO elevation in MCC and splenium	Menstrual cycle phase monitoring
**Animal Model**	**Guneykaya, 2023**	Neuroligin-4 KO mouse (C57BL/6J)	32 mice (8 per group for density)	16M / 16F	13 weeks (Young adult)	Hippocampus (CA3 region)	Proteomics, phagocytosis, P2RY12 current	Identified male-specific microglial state with metabolic and phagocytic impairments	Rescue effect of 17β-estradiol in males
**Animal Model**	**Dalton, 2024**	C58/J mouse (sociability focus)	28 mice (snRNA N=4; Bulk N=24)	snRNA: 2M / 2F; Bulk: 12M / 12F	7–8 weeks (Young adult)	Amygdala	snRNA-seq; homeostatic markers (Trem2, Cx3cr1)	Link between sociability deficits and reduced microglial homeostatic marker expression	Strain differences (C58/J vs C57BL/6J)
**In Vitro Functional Genomics**	**Teter, 2025**	CRISPRi screen in human hiMGL	Screen: 102 ASD risk genes	NA	NA	hiPSC-derived microglia	FACS-based synaptic pruning; motility; endocytosis	Identified ADNP as a key modifier of microglial synaptic pruning and endocytosis	In vitro iTF-Microglia system limitations
**Review / Perspective**	**Trujillo Villarreal, 2021**	NA	NA	NA	NA	NA	NA	Primed microglia in obesity disrupt mesocorticolimbic circuits	Focus on dopamine circuit E/I balance
**Mouse Model +**	**Wu, 2024**	SCN2A-	Mouse: n=8–10	Both sexes	Mice: 1–50 days &	Neocortex,	Spine density;	Microglial over-	PLX3397 microglial

**Organoid**		deficient model	per group; Organoids: NR	; exact M/F NR	85–95 days; Organoids: 150 days	Hippocampus, brain organoids	C3 cascade; PLX3397 ablation	pruning of post-synapses via C3 cascade leads to reduced transmission	ablation reverses phenotype
**Animal Model**	**Tian, 2024**	VPA rat model (prenatal exposure)	10 rats (5 male per group for behavior)	10M / 0F	PND 7, PND 28, PND 35	Prefrontal cortex (PFC)	TREM2/DAP12; P38 MAPK pathway	TREM2 improves synaptic development by inhibiting the P38 MAPK signaling pathway	In vitro rat primary microglia validation
**Animal Model**	**Wang, 2025**	Microglial TREM2 deficiency	29 mice (behavioral cohort)	29M / 0F	3–4 weeks (Young adult)	Neocortex (Layer 2/3)	Kv1.3 activity; mEPSCs/mIPSCs; TNF-α/IL-1β	TREM2 deficiency increases Kv1.3 activity, causing E/I imbalance and hyperactivity	Pharmacological Kv1.3 inhibition (PAP-1) rescue
**Animal Model**	**Lu, 2025**	TREM2 knockdown (AAV)	48 rats (16 per group x 3 groups)	48M / 0F	PND 14 to PND 42	Prefrontal cortex (PFC)	Iba1-positive/CD68 markers; RA/RARα signaling	TREM2-RA/RARα signaling disruption leads to aberrant microglial synaptic pruning	Oral RA supplementation rescue effect
**Review / Perspective**	**Boller, 2025**	NA	NA	NA	NA	NA	NA	CACNA1C genetic risk interacts with adaptive immune memory in neuroinflammation	Focus on L-type Ca2+ channels (CaV1.2)
**Review / Perspective**	**Bilbo, 2018**	NA	NA	NA	NA	NA	NA	Non-infectious MIA (e.g., air pollution) triggers microglial priming and ASD risk	Focus on the "multiple-hit" hypothesis

**Review / Perspective**	**Maldonado-Ruiz, 2019**	NA	NA	NA	NA	NA	NA	Maternal nutritional programming programs central microglial activation in MIA	Link between obesity and synaptic inputs
**Animal Model**	**Wang YC, 2023**	HFD × Cc2d1a cKO mouse	198 total animals	198 M / 0F	PND 21 to PND 84	Hippocampus (CA1)	Iba1-positive cell density; activated numbers	High-fat diet exacerbates ASD-like behavior via CA1 microglial activation	Minocycline treatment reverses behavioral deficits
**Human Postmortem Transcriptomics**	**Mou, 2022**	Human SVZ postmortem study	32 donors (16 ASD / 16 Ctrl)	ASD: 12M / 4F; Ctrl: 13M / 3F	ASD: 21.94 ± 9.28 years; Ctrl: 21.00 ± 3.58 years	SVZ, Septum pellucidum, Caudate nucleus	C4 mRNA/protein; Ki67 cell density	Association of complement C4 with neuroimmune abnormalities and neurogenesis in the SVZ	SVZ-specific focus; PMI and RIN controlled
**Review / Perspective**	**Jäntti, 2026**	NA	NA	NA	NA	NA	NA	Astrocyte-microglia crosstalk regulates E/I balance via mTOR and complement	Focus on human-relevant iMGL models
**Review / Perspective**	**Lyamtsev, 2025**	NA	NA	NA	NA	NA	NA	Male microglia are more prone to pro-inflammatory responses, favoring early ASD manifestation	Explores the "inflammaging" timeline
**Animal Model**	**Zhang Q, 2024**	VPA-induced mouse model (C57BL/6)	8 mice (4 Ctrl / 4 VPA)	4M / 4F (2M / 2F per group)	8–9 weeks (Young adult)	Hippocampus	snRNA-seq; TNF/NF-κB pathways	Males show more significant up-regulation of inflammatory pathways in hippocampa	45,693 single nuclei analyzed across 12 types

								l microglia	

Abbreviations: STG, superior temporal gyrus; PFC, prefrontal cortex; ACC, anterior cingulate cortex; MCC, midcingulate cortex; OFC, orbitofrontal cortex; PCC, posterior cingulate cortex; CC, corpus callosum; SVZ, subventricular zone; PND, postnatal day. WT, wild type; KO, knockout; VPA, valproic acid (prenatal exposure model). *NR*, not reported; *NA*, not applicable.

Table S2. Modality-specific confounders and interpretation safeguards

Purpose: Provide a practical checklist of common confounders and safeguards when interpreting ASD microglia signals across modalities. This table is intended to aid cautious cross-modality interpretation and to contextualize differences in evidence strength.

Modality	Common confounders	Practical safeguards	Interpretation notes
Bulk postmortem transcriptomics	Cell composition; PMI/RIN; cause of death; agonal state; medication; brain bank batch effects; region sampling	Deconvolution / cell-type markers; include PMI/RIN covariates; sensitivity analyses; replicate across cohorts; region-stratified analyses	Avoid equating bulk immune modules with microglial state shifts; emphasize association vs mechanism
snRNA-seq / scRNA-seq (postmortem)	Ambient RNA; doublets; cell calling differences; donor imbalance; low microglia counts; dissociation bias	Standard QC; doublet removal; donor-aware models; composition-adjusted DE; validate with spatial/IHC where possible	Report N donors and microglia per donor; avoid overinterpreting rare clusters without replication
Spatial transcriptomics / in situ	Resolution limits; spot mixing; segmentation errors; tissue quality; region selection bias	Cell-type deconvolution per spot; confirm with marker IHC/IF; analyze multiple ROIs; replicate patterns across samples	Use spatial context to support niche claims (neurovascular, layer-specific, white/gray matter)
Histology / IHC / IF / morphology	Antibody specificity; fixation differences; quantification bias; limited sampling; small N	Validated antibodies; blinded quantification; multiple markers; standardize imaging/thresholding; report effect sizes with N	Morphology is not synonymous with activation; interpret with molecular/functional readouts
TSPO PET / neuroimmune imaging	TSPO polymorphisms; tracer differences; non-microglia sources; partial volume; medication; region-of-interest choices	Genotype correction; harmonize tracers/analysis; sensitivity to ROI definitions; interpret as glia-associated tone, not microglia state	Treat TSPO as an imperfect glia-associated proxy and interpret it alongside other modalities (e.g., CSF, postmortem data, genetics).
Genetics (GWAS, rare variants)	Pleiotropy; cell-type assignment uncertainty; developmental context; linkage disequilibrium; ancestry	Cell-type specific enrichment with multiple methods; integrate with functional assays; cautious causal language	Genetic enrichment supports plausibility, not directionality of microglial effects
ASD model animals (mouse, etc.)	Model validity; strain effects; sex and age; housing; assay variability; microglia ontogeny differences	Match developmental windows; include both sexes; replicate in independent cohorts; rescue experiments; blinded behavioral analyses	Be explicit about construct vs face validity; map findings to human-readable effectors

Human iPSC / organoids / assembloids	Maturation state; batch effects; cell-line variability; microglia integration; limited immune milieu	Isogenic controls; multiple clones/lines; standardized differentiation; functional readouts (engulfment, cytokines, synapse density)	Useful for perturbation-based inference but limited in capturing whole-brain niche context; interpret alongside in vivo developmental timing.

Table S3. Microglia-related states, pathways, and functional themes discussed in ASD literature

Purpose: This table summarizes recurrent microglia-related terminology used across ASD studies, including states, pathways, and functional themes. It is provided as a literature-oriented terminology summary rather than a fixed mechanistic framework.

Microglia-related state / pathway / theme	Category	Functional relevance	ASD-relevant evidence base
Homeostatic microglia	Baseline state	Physiological brain surveillance and maintenance	Characterized by markers such as *P2RY12* and *TMEM119*; reported to be reduced in human ASD cortex and specific animal models (e.g., C58/J, *Mecp2*-null).
asdM16 / mod5 innate immune module	Immune signaling program	Innate immune activation and type I interferon response	Identified via bulk transcriptomics co-expression analysis; enriched for M2-state markers and antiviral signaling genes in human postmortem ASD tissue.
MG2 (Reactive microglia cluster)	Immune communication program	Reactive glial signaling and inflammatory-state enrichment	Identified in large-scale human snRNA-seq cohorts as a reactive cluster significantly increased in ASD gray matter, associated with TFs such as *IRF8* and *JUND*.
Phagocytic–lysosomal capacity	Phagocytosis / clearance program	Synaptic pruning and debris removal	Supported by ASD model studies linking microglial engulfment/clearance biology to pruning-related phenotypes (e.g., *SCN2A* deficiency, *TREM2* models).
SPP1+ microglia state	Remodeling-associated state	Candidate phagocytosis- and remodeling-associated state	Correlated with focal reactivity and synaptic contact; identified as a distinct sub-cluster in multimodal genomics data linked to ASD risk targets.
TREM2–APOE lipid handling program	Immunometabolic program	Discussed as an immunometabolic module with emerging relevance in ASD-related models	Linked to microglial metabolic fitness and transition to activated states; loss of *TREM2* impairs pruning capacity and E/I balance in multiple ASD models.
PI3K–Akt–mTOR signaling	Immunometabolic program	Regulation of microglial metabolism and protein synthesis	Shared pathway identified across high-confidence risk genes (e.g., *PTEN*, *TSC1/2*); controls microglial clearance capacity and proliferative states.
Complement cascade (C1q, C3, C4)	Immune signaling program	Molecular tagging for microglial synaptic engulfment	Upregulated in human ASD SVZ and cortex; loss-of-function models (*SCN2A*, *MEF2C*) show complement-associated pruning abnormalities and connectivity

			deficits.
CX3CL1–CX3CR1 axis	Immune communication program	Neuron–microglia chemotactic and functional interaction	Critical for microglial recruitment and maturation of synapses; deficiency leads to transient social deficits and reduced functional connectivity in mouse models.
Prenatal priming / MIA response	Developmental state/program	Early-life sensitivity to environmental immune triggers	Examined in VPA and Poly(I:C) models; used to study how maternal inflammation may prime fetal microglia toward persistent inflammatory bias across development.
TSPO imaging proxy	Imaging proxy	In vivo proxy for glia-associated neuroimmune status	Investigated through human PET imaging; shows regional distribution shifts (increased in MCC/splenium; decreased in other regions) in young adult ASD cohorts

Supplementary Figures

Figure S1. Literature identification and evidence organization workflow.

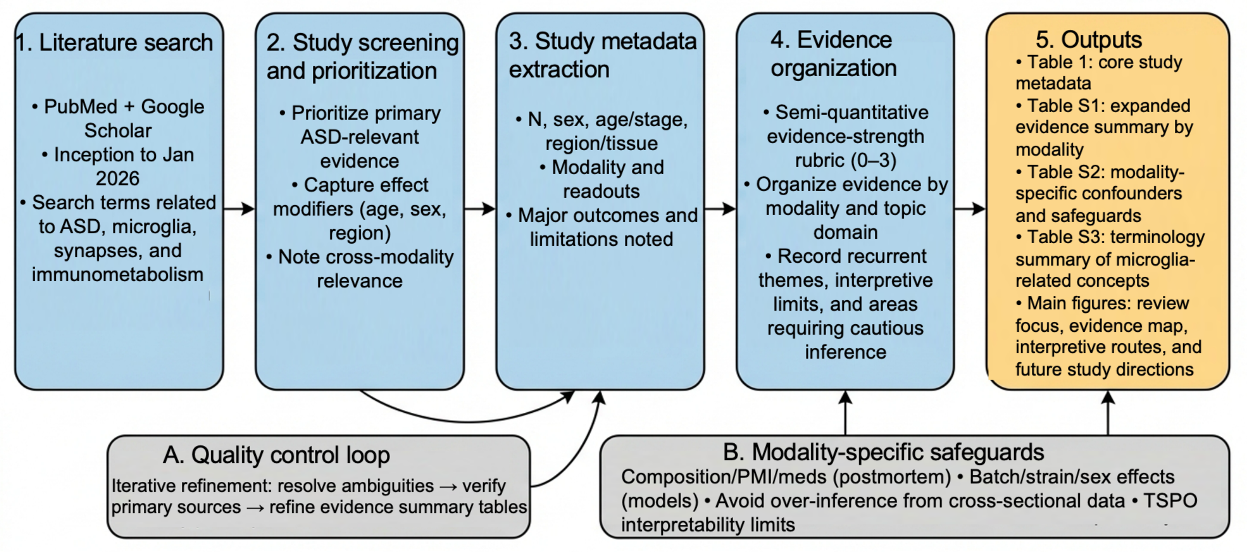

Schematic overview of how studies were identified, screened, extracted, and organized across modalities for this Review. The workflow summarizes literature search, prioritization of ASD-relevant evidence, extraction of study-level metadata, semi-quantitative organization of evidence by modality and topic domain, and generation of supplementary tables and summary figures. This figure is intended as a methodological aid for evidence organization rather than a mechanistic framework.
